# Smoking-related cancer in military veterans: retrospective cohort study of 57,000 veterans and 173,000 matched non-veterans

**DOI:** 10.1186/s12885-016-2347-5

**Published:** 2016-05-14

**Authors:** Beverly P. Bergman, Daniel F. Mackay, David Morrison, Jill P. Pell

**Affiliations:** Institute of Health and Wellbeing, University of Glasgow, Glasgow, G12 8RZ UK

**Keywords:** Veterans, Military, Smoking, Lung cancer, Smoking-related cancer, Retrospective cohort study

## Abstract

**Background:**

Serving military personnel are more likely to smoke, and to smoke more heavily, than civilians. The aim of our study was to examine whether veterans have an increased risk of a range of smoking-related cancers compared with non-veterans, using a large, national cohort of veterans.

**Methods:**

We conducted a retrospective cohort study of 57,000 veterans resident in Scotland and 173,000 age, sex and area of residence matched civilians. We used Cox proportional hazard models to compare the risk of any cancer, lung cancer and other smoking-related cancers overall, by sex and by birth cohort, adjusting for the potential confounding effect of socioeconomic deprivation.

**Results:**

Over a mean of 29 years follow-up, 445 (0.79 %) veterans developed lung cancer compared with 1106 (0.64 %) non-veterans (adjusted hazard ratio 1.16, 95 % confidence intervals 1.04–1.30, *p* = 0.008). Other smoking-related cancers occurred in 737 (1.31 %) veterans compared with 1883 (1.09 %) non-veterans (adjusted hazard ratio 1.18, 95 % confidence intervals 1.08–1.29, *p* < 0.001). A significantly increased risk was observed among veterans born 1950–1954 for lung cancer and 1945–1954 for other smoking-related cancers. The risk of lung cancer was decreased among veterans born 1960 onwards. In comparison, there was no difference in the risk of any cancer overall (adjusted hazard ratio 0.98, 95 % confidence intervals 0.94–1.01, *p* = 0.171), whilst younger veterans were at reduced risk of any cancer (adjusted hazard ratio 0.88, 95 % confidence intervals 0.81–0.97, *p* = 0.006).

**Conclusions:**

Military veterans living in Scotland who were born before 1955 are at increased risk of smoking-related cancer compared with non-veterans, but younger veterans are not. The differences may reflect changing patterns of smoking behaviour over time in military personnel which may, in turn, be linked to developments in military health promotion policy and a changing military operational environment, as well as to wider societal factors.

## Background

In 1950, Doll and Hill published epidemiological evidence of the link between smoking and lung cancer which had been postulated by Müller in 1939 [1, 2]. By 2004 clear evidence had been established of an association between smoking and many other cancers including larynx, oropharynx, oesophagus, stomach, bladder and, more recently recognised, colon and rectum [3]. Tobacco smoking has been shown to be responsible for 85 % of lung cancer in men and 80 % in women in the UK, whilst 23 % of all cancer in men and 15 % in women can be attributed to smoking [4]. Studies of military smoking habits have consistently shown that soldiers have a higher prevalence of smoking, and smoke more heavily, than civilians [5, 6]. Data on smoking in veterans are more sparse but US studies have shown that they also smoke more than non-veterans [7, 8]. To the best of our knowledge, no studies have previously been published on smoking prevalence among UK veterans.

In the worldwide military context, there has been much anxiety about the carcinogenic potential of substances such as chemical warfare agents, ionising radiation, defoliants, industrial chemicals and the smoke from the destruction of industry and infrastructure to which military personnel may have been exposed in the course of their duties [9]. Although risk assessment is now routine for known military occupational hazards [10], the nature of warfare inevitably gives rise to unexpected hazard exposures. Personal lifestyle choices such as smoking during service may also increase the risk of cancer in this population [11]. The long latency time for smoking-related cancers [12] means that most cases will present after leaving service. A long-term follow-up of US veterans of World War 2 and the Korean War found an excess of lung cancer and attributed around half of the excess risk to “military-induced smoking” [13], and a 50-year follow-up of Australian Korean War veterans [14] also found that they were at increased risk of lung cancer.

The Scottish Veterans Health Study provided an opportunity to examine rates of all cancer and smoking-related cancer in a large cohort of UK military veterans, irrespective of exposure to conflict, in comparison with civilians with no record of service, in order to determine whether veterans are at increased risk.

## Methods

The Scottish Veterans Health Study is a retrospective cohort study of all 56,570 military veterans resident in Scotland who were born between 1945 and 1985 and who were registered with National Health Service (NHS) Scotland both before and after service, and a comparison group of 172,753 individuals with no record of service matched 3:1 for age, sex and postcode sector of residence (mean population 5000). The study cohort and methods have been described elsewhere [15]. Demographic data obtained from electronic NHS registration records were linked at an individual level to routine hospital admissions data (Scottish Morbidity Record SMR01), cancer registration data (SMR06) and death certificates to provide information on first episode of smoking-related cancer (hospitalisation or death) and all-cause death. Individual-level prescribing data were obtained from the Prescribing Information System for a limited range of drugs including nicotine replacement therapy (NRT).

The electronic NHS record provided dates of entering and leaving the Service for veterans. The maximum period of follow-up was from 1 January 1981 (or date of leaving the Service, for veterans, if later) to 31 December 2012. The data extract was pseudo-anonymised and approval for the study was granted by the Privacy Advisory Committee of the Information Services Division of NHS Scotland.

In Scotland, there are 6505 datazones, based on postcode of residence, with a mean population of 800. The Scottish Index of Multiple Deprivation (SIMD) for each datazone is derived from information on income, employment, health, education (including skills and training), housing, crime and access to services (http://www.scotland.gov.uk/Topics/Statistics/SIMD). The SIMD has been used to derive quintiles of regional socioeconomic status (SES) for the Scottish population; ranging from 1 (most deprived) to 5 (least deprived). We used postcode of residence to categorize the cohort participants according to these quintiles.

‘Any cancer’ was defined as ICD9 codes 140–209 and 230–234, and ICD10 codes C00-C97 and D00-09. ‘Smoking-related cancers’ were defined as lung (ICD9 162, ICD10 C34), stomach (ICD9 151.9, ICD10 C16), oesophagus (ICD9 150, ICD10 C15), oropharynx and larynx (ICD9 146, 161, ICD10 C01, C09, C10, C32), bladder (ICD9 188, ICD10 C67) and kidney (ICD9 189.0, ICD10 C64), at any position in the record. Cox proportional hazard models were used to examine the association between veteran status and risk of any cancer, lung cancer and other smoking-related cancer (stomach, oesophagus, oropharynx and larynx, bladder, kidney; combined and separately), using age as the time dependent variable, first smoking-related cancer as the failure time and death (if no smoking-related cancer) as the censor time. The a priori rejection level was set at 0.05. Cox proportionality assumptions were tested using methodology based on Schoenfeld residuals [16]. The log-likelihood test was used to test for interactions with sex and birth cohort. A landmark analysis was performed using age 40 years as the starting point. The models were run univariately and then repeated adjusting for the potential confounding effect of regional SES. The analyses were repeated stratifying by grouped year of birth in 5-year categories to examine potential birth cohort effects, and by length of service in two categories (less than 3 years and 4 or more years) to examine the effect of failure to complete the minimum term of military engagement (Early Service Leavers (ESL)). The cumulative hazard for lung cancer was presented graphically as a Nelson-Aalen plot. All analyses were performed using Stata v12.1 (©1985-2011 StataCorp).

## Results

After data cleansing, 56,205 (99.3 %) veterans and 172,741 (99.9 %) non-veterans were included in the analysis. Of the 56,205 veterans included in the study, 5235 (9.2 %) were women, reflecting the gender balance of the Service population. The mean period of follow-up was 29.3 years, with a total of 6.7 million person-years of follow-up among veterans and non-veterans combined.

There were 3588 (6.38 %) cases of any cancer in the 56,025 veterans during the period of follow-up, compared with 11,560 (6.69 %) cases in the 172,741 non-veterans. The difference was not statistically significant overall. Among those born prior to 1960 there was no significant difference between veterans and non-veterans, whereas among those born from 1960 onwards, there was a significantly lower risk of any cancer among veterans (Table 1).

**Table 1:** Cox proportional hazard model of the association between veteran status and risk of all cancers and smoking-related cancers

	Univariate			Multivariate^a^		
Cancer	HR	95 % CI	*P* value	HR	95 % CI	*P* value
All cancers	0.98	0.94–1.01	0.197	0.98	0.94–1.01	0.171
Birth year < 1960	1.00	0.96–1.04	0.976	0.99	0.96–1.04	0.873
Birth year 1960 onwards	0.88	0.80–0.96	0.004	0.88	0.81–0.97	0.006
Lung cancer	1.22	1.09–1.36	0.001	1.16	1.04–1.30	0.008
Other smoking-related cancers	1.20	1.10–1.31	<0.001	1.18	1.08–1.29	<0.001
Oropharyngeal/laryngeal cancer	1.25	1.09–1.44	0.002	1.21	1.05–1.39	0.009
Oesophageal cancer	1.26	1.04–1.52	0.019	1.23	1.02–1.49	0.032
Stomach cancer	1.27	1.00–1.61	0.052	1.24	0.98–1.58	0.074
Kidney cancer	0.98	0.79–1.22	0.863	0.97	0.78–1.21	0.809
Bladder cancer	1.21	0.98–1.49	0.082	1.21	0.98–1.49	0.077

*HR* hazard ratio, *CI* confidence interval

^a^adjusted for Scottish Index of Multiple Deprivation

**Fig. 1:**
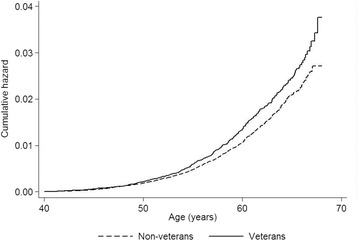
Nelson-Aalen cumulative hazard plot showing risk of lung cancer by veteran status

**Fig. 2:**
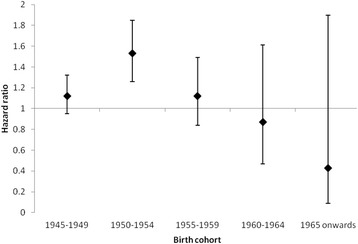
Hazard ratio for lung cancer in veterans referent to non-veterans by birth cohort

A total of 445 (0.79 %) veterans had a diagnosis of lung cancer compared with 1106 (0.64 %) non-veterans, equating to an incidence of 3.47 per 10,000 person-years among veterans compared with 2.04 among non-veterans, rate ratio (RR) 1.70, 95 % CI 1.52–1.90. For veterans aged over 50 years, there were 12.29 cases per 10,000 person-years compared with 10.02 for non-veterans, RR 1.23, 95 % CI 1.08–1.39. The Cox proportional hazard model for men and women combined showed a statistically significantly increased risk of lung cancer among veterans both in the univariate model, hazard ratio (HR) 1.22, 95 % CI 1.09–1.36, *p* = 0.001 (Fig. 1) and after adjusting for SES, HR 1.16, 95 % CI 1.04–1.30, *p* = 0.008. The hazard ratios were increased for both men and women but were only statistically significant for men (Table 1). Tests for non-proportionality of hazard were non-significant. Testing for interaction was non-significant for sex but significant for birth cohort. When stratified by birth cohort, the increased risk was statistically significant for veterans born 1950–1954 (Fig. 2) and there was a non-significant increase for veterans born 1945–1949 and 1955–1959 (Table 2). There was a non-significant decrease in risk of lung cancer for veterans born from 1960 onwards compared with non-veterans (Fig. 2).

**Table 2:** Cox proportional hazard model of the association between veteran status and risk of lung cancer

		Univariate			Multivariate^a^		
Subgroup		HR	95 % CI	*P* value	HR	95 % CI	*P* value
Overall		1.22	1.09–1.36	0.001	1.16	1.04–1.30	0.008
Sex	Men	1.23	1.09–1.38	0.001	1.17	1.04–1.31	0.008
	Women	1.09	0.72–1.66	0.671	1.07	0.70–1.62	0.760
Birth year	1945–1949	1.12	0.95–1.32	0.174	1.07	0.91–1.27	0.399
	1950–1954	1.53	1.26–1.85	<0.001	1.44	1.19–1.74	<0.001
	1955–1959	1.12	0.84–1.49	0.440	1.06	0.80–1.42	0.675
	1960–1964	0.87	0.47–1.61	0.664	0.87	0.47–1.61	0.656
	1965 onwards	0.43	0.10–1.90	0.267	0.45	0.10–1.96	0.287
ESL status	ESL	1.46	1.22–1.75	<0.001	1.32	1.10–1.58	0.002
	Non-ESL	1.10	0.96–1.26	0.173	1.08	0.99–1.22	0.291

*HR* hazard ratio, *CI* confidence interval, *ESL* Early Service Leaver – left service before completing initial term of engagement

^a^adjusted for Scottish Index of Multiple Deprivation

**Fig. 3:**
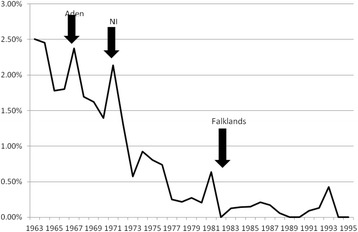
Crude incidence of lung cancer in veterans by year of entry to the Armed Forces, showing major military deployments

Early Service Leavers (ESL) showed a greater increase in risk of lung cancer compared with non-veterans (adjusted HR 1.32, 95 % CI 1.10–1.58, *p* = 0.002) than veterans who had completed at least the minimum term of military engagement (adjusted HR 1.08, 95 % CI 0.99–1.22, *p* = 0.291) (Table 2). When analysed by year of entry to service (crude percentage of each year intake who have developed lung cancer), there were peaks of incidence corresponding with entry during, or immediately prior to, major periods of military operational activity (Fig. 3). The small peak in 1993 does not coincide with a deployment; it represents only 2 cases, at a period of low recruitment.

There were 737 (1.31 %) other smoking related cancers in veterans compared with 1883 (1.09 %) in non-veterans. In men, the numbers were 713 (1.40 %) and 1796 (1.18 %) respectively and in women they were 24 (0.46 %) and 87 (0.42 %) respectively. There was an incidence of 5.66 per 10,000 person-years for other smoking-related cancers in veterans compared with 3.47 in non-veterans, RR 1.63, 95 % CI 1.49–1.77. In the Cox proportional hazard models, including both men and women, veterans were at significantly higher risk in both the univariate (HR 1.20, 95 % CI 1.10–1.31, *p* < 0.001) and multivariate analyses (HR 1.18, 95 % CI 1.08–1.29, *p* < 0.001). The hazard ratios were increased for both men and women but were only statistically significant for men (Table 3). The risk was increased for all smoking-related cancers except kidney, although it only achieved statistical significance for oropharyngeal cancer and oesophageal cancer (Table 1). Tests for non-proportionality of hazards, and for interaction for sex and birth cohort, were non-significant but analysis by birth cohort was performed in view of the results for lung cancer. This showed a significance increase in risk for veterans born 1945–1954. For veterans aged over 50, there were 17.44 cases per 10,000 person-years compared with 14.92 for non-veterans (RR 1.18, 95 % CI 1.07–1.31). There was no difference in risk for veterans born from 1955 onwards (Table 3). ESL were at only slightly increased risk of other smoking-related cancers compared with non-veterans than were veterans who had completed the minimum engagement (Table 3).

**Table 3:** Cox proportional hazard model of the association between veteran status and risk of other smoking-related cancers^a^

		Univariate			Multivariate^b^		
Subgroup		HR	95 % CI	*P* value	HR	95 % CI	*P* value
Overall		1.20	1.10–1.31	<0.001	1.18	1.08–1.29	<0.001
Sex	Men	1.18	1.08–1.29	<0.001	1.16	1.06–1.27	0.001
	Women	1.21	0.75–1.96	0.424	1.21	0.75–1.95	0.430
Birth year	1945–1949	1.29	1.12–1.48	<0.001	1.26	1.10–1.43	0.001
	1950–1954	1.29	1.10–1.50	0.002	1.25	1.07–1.46	0.005
	1955–1959	0.99	0.79–1.23	0.899	0.97	0.78–1.21	0.798
	1960–1964	1.04	0.75–1.43	0.817	1.05	0.76–1.45	0.774
	1965 onwards	0.98	0.53–1.83	0.959	0.99	0.53–1.84	0.977
ESL status	ESL	1.26	1.08–1.46	0.003	1.20	1.03–1.39	0.017
	Non-ESL	1.18	1.07–1.32	0.001	1.18	1.06–1.31	0.002

*HR* hazard ratio, *CI* confidence interval, *ESL* Early Service Leaver – left service before completing initial term of engagement

^a^stomach, oesophagus, oropharynx and larynx, bladder, kidney

^b^adjusted for Scottish Index of Multiple Deprivation

Among older study participants (born 1945–1959), veterans were more likely to have received a prescription for nicotine replacement therapy than non-veterans, adjusted odds ratio (OR) 1.41, 95 % CI 1.35–1.48, *p* < 0.001. For younger participants (born 1960 onwards), there was a non-significant reduction in the likelihood of receiving a prescription for NRT among veterans, adjusted OR 0.96, 95 % CI 0.91–1.02, *p* = 0.159.

## Discussion

The Scottish Veterans Health Study demonstrated that although overall rates of cancer showed no differences between veterans and non-veterans, the veterans were at increased risk of developing lung cancer and other smoking-related cancers compared with civilians with no record of service matched for age, sex and area of residence. The increase was only apparent for veterans born before 1960, and was greatest in those born between 1950 and 1954. For veterans born from 1960 onwards there was a non-significant reduction in risk in lung cancer compared with non-veterans, whilst the reduction in risk for all cancers in this group was statistically significant. Both male and female veterans demonstrated an increased risk of smoking-related cancer, although the results were only statistically significant for men. There were peaks of incidence of lung cancer associated with having joined the Armed Forces during or immediately prior to a period of heightened operational activity. The increase in risk was highest in those with the shortest service. Our finding of increased prescribing of NRT for the older veterans compared with non-veterans suggests that smoking was more prevalent in older veterans and may account for the higher prevalence of smoking-related cancers although as we had no data on occupational exposures, other contributory factors cannot be ruled out.

The increased risk of smoking-related cancers in veterans who entered the Armed Forces in the 1960s and early 1970s is consistent with earlier studies on smoking prevalence in military personnel. In the first formal study of UK military smoking rates, Richards and Crowdy reported that the prevalence of smoking among junior soldiers aged 15–19 years (born 1940–1944) was 17-20 % higher than the general population prevalence quoted by the Tobacco Manufacturers’ Standing Committee. The prevalence of regular smoking ranged from 51 to 58 % at age 15 years to 79–80 % at age 18–19 years, with the greatest increase taking place during the 16th year [17]. The soldiers also smoked more heavily; in 1969, 65 % of male Army smokers aged 20–34 smoked more than 20 cigarettes per day compared with 53 % of civilian smokers [18]. Since then, smoking prevalence in the UK Armed Forces has fallen, even before the implementation of formal military smoking cessation initiatives in 2001, and by 1989 only 38.7 % of Service personnel were current smokers although 58.8 % were ever-smokers [6]. The UK Defence Dental Agency conducted a survey of 10,531 military personnel who enlisted in 1998–9 and found that there was a higher prevalence of smoking amongst the 3596 Army personnel at 44.7 % on enlistment, compared with 34.1 % for the Royal Navy and 30.5 % for the Royal Air Force. The cohort was followed up in 2002 when 47.8 % of the Army personnel who were still serving were smokers [19], much higher than the general population prevalence of smoking of 26 % [20]. Few claimed to have started smoking for the first time since enlistment and the majority of the ‘new’ smokers were those who had restarted after having been smokers prior to enlistment. Army smoking rates were highest in infantry soldiers and lowest among officers [19]. A cross-sectional study of male service personnel in 2004 found that the overall prevalence of smoking had fallen below the civilian rate although personnel in the Army and of lower rank and educational attainment were more likely to report being smokers [21].

Higher rates of smoking in military personnel than civilians have also been described in a number of other armed forces. In 1986, Schei examined changes in smoking behaviour as a result of military service in 2105 Norwegian conscripts aged 18–25 years. Of those who were non-smokers on enlistment, 7.8 % had started smoking during their service, whilst of those who were already smokers, 55.7 % reported smoking more heavily during their military service. The prevalence of daily smoking was 51 % among army conscripts during service compared with a population prevalence of 37.2 % in men aged 19–21 and 35.4 % in men aged 22–24 years in the general population. Living in dormitories where smoking was permitted resulted in 90.8 % being exposed to environmental tobacco smoke [22]. Feigelman summarised a number of studies conducted for the US Department of Defence in the 1980s which also showed a higher rate of smoking in soldiers than in civilians; in 1982 51 % of military personnel smoked compared with 36 % of men and 29 % of women in the US adult population and although the military rate had fallen to 41 % by 1988, it remained higher than the general adult population rate of 27 % [23].

We were unable to obtain data on smoking prevalence in our cohort and therefore used NRT prescribing as a novel proxy measure for relative prevalence in veterans and non-veterans. Although fewer studies have been conducted on smoking prevalence in veterans than in serving personnel, a consistent pattern of higher prevalence persisting into post-service life has been demonstrated, predominantly in the US. The first major study to compare smoking rates in veteran and non-veteran civilian populations was the 1987 US National Medical Expenditure Survey, a questionnaire-based study which gathered data from 36,400 people in 15,000 US households; 3372 male and female veterans were compared with 18,606 male and female non-veterans. Seventy-seven per cent of veterans were ever-smokers of cigarettes compared with 47 % of non-veterans. Thirty-five per cent of veterans were current cigarette smokers compared with 28 % of non-veterans, the mean age at smoking cessation for those who had quit being 39 years for veterans and 40 years for non-veterans [8]. The findings are similar to the results of an analysis of the US National Health Interview Survey which found rates of ever-smoking of 74.2 % in veterans and 48.4 % in non-veterans, and rates of current smoking of 33.9 % in veterans and 27.7 % in non-veterans, with no significant differences in quit behaviour [24]. Brown analysed data from the telephone-based Centers for Disease Control (CDC) Behavioral Risk Factors Surveillance Study (BRFSS) for 2003–2007 covering 224,169 veterans, 93 % of whom were male, and found the age-adjusted period prevalence of current smoking was 27 % in veterans and 21 % in non-veterans [7].

Feigelman also examined data on 698 male veterans and 1505 male civilians gathered between 1977 and 1991 for the US General Social Survey and found that 47.6 % of veterans smoked compared with 40.7 % of civilians. The author concluded that higher rates of smoking in serving military personnel led to lifelong elevated rates of smoking, and that this also generated an environment in which spouses and children of military personnel were more likely to smoke, thus increasing the long-term adverse public health impact of military smoking [23]. A study of Australian male Korean War veterans (mean age 75 years) more than 50 years after the conflict showed that 12 % were current smokers and 79 % were ever-smokers, compared with 7.0 and 60 % respectively for controls drawn from the elderly male Australian population [25].

The overall increased risk of lung cancer and other smoking-related cancers in veterans in our study is also consistent with the findings of other studies. In a major study of the long-term health of US World War II and Korean War veterans followed up for 20–50 years, Bedard et al. calculated an implied mortality rate for lung cancer of 2.2 per 1000 for veterans compared with 1.2 per 1000 for non-veterans and estimated that 35–58 % of the excess veteran lung cancer deaths were attributable to military-induced smoking [13]. Watanabe et al. noted a statistically significantly increase in mortality from lung cancer and laryngeal cancer in Vietnam veterans compared with veterans who had not served in Vietnam [26]. Rogot reported higher mortality for cancer of the lung, larynx, stomach, oesophagus, bladder, kidney and other sites in smokers than non-smokers in a cohort of 294,000 US veterans followed up from 1954 to 1969, but did not undertake a comparison with rates in non-veterans [27]. Further follow-up of the same cohort to 1980 demonstrated continuing increased risk for ever-smokers compared with never-smokers [28]. A study of mortality in 1789 veteran British Army cooks followed up from 1974 to 1989 found that lung cancer mortality was elevated in comparison with the national population (SMR 1.82, 95 % CI 1.25–2.57). The authors attributed part of the difference to smoking although they were unable to rule out occupational factors. An internal control group of 1310 former Royal Army Pay Corps personnel also showed an increased risk of lung cancer although this did not reach statistical significance (standardised mortality ratio (SMR) 1.38, 95 % CI 0.88–2.07) [29].

The reduced risk for cancer overall in younger veterans in our study is consistent with an Australian cohort study which followed up veterans of the first Gulf War from 1991 to 1998 and found a standardised incidence ratio of 71 (95 % CI 37–137) for all cancers compared with the general population. The veterans had a mean age of 27 years at entry to the study in 1991 [30]. Follow-up of 21,432 UK Falklands veterans (86 % of the total) from 1982 to 2012 showed a reduced risk of mortality from any cancer compared with the general population (SMR 70, 95 % CI 64–77), similar to the SMR for neoplasms in the Gulf cohort and matched ‘Era’ comparison group of non-deployed personnel followed up since the 1991 Gulf War (SMR 60, 95 % CI 54–67 and SMR 66, 95 % CI 60–73 respectively). In both Gulf and Falklands groups, the commonest cancer site was bronchus/lung [31, 32].

Our finding of a higher incidence of smoking-related cancer in older veterans who joined the services at times of major operational activity is consistent with reports of increased smoking on operations. The US military Millennium Cohort Study, a 21-year prospective study, has demonstrated that operational deployment is associated with an increased risk of both smoking initiation in previous never-smokers and a resumption of smoking in ex-smokers, and that the risk is further increased with exposure to combat [33]. There is good evidence that US troops returning from Afghanistan are smoking heavily [34]. A small study in US troops serving in Afghanistan in 2011 found that 62 % of deployed personnel smoked and that 29 % had increased their tobacco usage whilst deployed. The authors used cigarette sales figures to derive an estimate that tobacco use for deployed personnel was approximately 3 times the US national average. Predisposing factors for smoking identified by the troops included stress (35 %), boredom (30 %) and addiction (20 %) [35]. In an unpublished thesis, Hodgson found that soldiers valued smoking as one of the few activities which they controlled, and that “smoke breaks” provided an important break from responding to military orders [36].

In our study, the oldest veterans commenced their service in 1960 as junior soldiers, although they would not have become eligible for deployment until the age of 18, in 1963. UK military operations in the 1960s included Aden (December 1963–November 1967) and Northern Ireland (from August 1969), so those who joined from the mid-1960s (born 1950 onwards) and completed training were highly likely to have seen operational service. Operations in Northern Ireland continued through the 1970s and did not conclude until 2007, although there were major shifts in operational tempo over that period, the earliest period (up to and including 1972) generally having been the most intense. Our finding of the highest risk of smoking-related cancer in veterans born 1950–1954 suggests that this early period of intense operational activity was associated with increased rates of smoking. This is borne out by the pattern of peaks of incidence of lung cancer in those who joined for service during or shortly before the periods of high-intensity operations. Taken together with our finding that ESL demonstrated a greater increase in risk than those with longer service, there is a strong indication that the most junior personnel were at highest risk, consistent with Lynch’s conclusion that smoking habits, and hence risk profile for smoking-related ill-health, are determined in the first few years of Army life [37]. It also suggests that their service may have been too short to benefit from military health promotion initiatives.

The Cold War (approximately 1947–1991) resulted in many people spending long periods engaged in training and exercises in Germany, where tobacco was readily available tax-free. By the late 1970s when the veterans born from 1960 were entering service, not only had there been changes in operational tempo but there was also an increasing emphasis on military health and fitness. The Falklands War (April–June 1982) involved around 28,000 UK military personnel but was a brief conflict and may have had a smaller impact on military smoking rates than the more sustained campaigns, although Fig. 3 provides evidence of a small increase in incidence of lung cancer in this group. It remains too early to detect any long-term impact on smoking-related cancer arising from British military deployments to the Gulf or Afghanistan with certainty although follow-up of the 1990–1991 Gulf War cohort to 2012 shows a statistically significantly lower number of deaths from lung cancer in those who deployed compared with the ‘Era’ controls who served at the same time but did not deploy [31]. This is consistent with evidence of a decline in use of cigarettes with deployment or exposure to combat in Iraq between 2002 and 2005 [38] but longer follow-up is required in order to evaluate the impact of this complex picture of smoking in relation to deployment.

In our study, the hazard ratios were statistically significant for increased risk of smoking-related cancer in veteran men but not in women. It is likely that small numbers of cases in women, who constituted only 9 % of the veteran study population, provided insufficient statistical power to demonstrate significance. Studies on smoking behaviour in serving UK military women are sparse, whilst none have been identified on smoking in UK female veterans. In a study of military healthcare workers deployed to Iraq, Boos and Croft reported no differences in smoking rates between men and women [39], whilst a US study reported that veteran women were significantly more likely than civilian women to smoke (19.4 % compared to 15.1 %, *p* < 0.001) [40]. Notwithstanding the paucity of data, there is some evidence that the health-related behaviour of serving military women is similar to that of men; Rona et al., in a study of alcohol misuse in UK armed forces personnel, reported that the pattern of results for men and women was similar [41].

The strengths of the present study are that it was based on a large cohort covering the whole of Scotland with 30 years follow-up. The cancer diagnosis was taken from hospital admission, cancer registration and death records, and is therefore likely to be both reliable and reasonably complete in respect of those events occurring within Scotland. The use of record linkage to analyse individual level data directly derived from health records allowed a robust cohort study design to be employed. The results were able to be matched or adjusted for potential confounders including sex, deprivation and geography. It was possible to do subgroup analysis by sex, and the ability to study birth cohort effects and incidence by year of entry to service contributes to the understanding of factors influencing veterans’ health. The use of a Nelson-Aalen plot at Fig. 1 allowed us to accommodate censored or incomplete data. The ability to analyse the data by length of service has provided an important insight into the long-term physical health of a large cohort of Early Service Leavers; whilst there has been some limited research on their mental health [42], their physical outcomes have not been documented prior to the Scottish Veterans Health Study, to the best of our knowledge.

A limitation of the study is the loss to follow-up of subjects due to migration away from Scotland, which cannot be quantified. There were no follow-up data prior to 1 January 1981 but since the oldest of the cohort were only aged 36 years at that date and the landmark entry point was age 40 years, and few smoking-related cancers would be expected to develop prior to that age, we considered the effect of immortal time bias to be negligible. As the oldest of the cohort was aged 67 years at the end of follow-up and 95 % were under age 65 years, many smoking-related cancers would not yet have developed by the end of the study period. For the veterans, we have not been able to link to military health records and thus cancers developing during service will not have been captured until medical retirement and return to NHS care. However as most personnel leave the Armed Forces by age 40 years, the landmark entry point will have minimised any risk of loss of information. No information was available on personal lifestyle risk factors which could have modified the risk of cancer. Therefore, receipt of an NRT prescription was used as a proxy for relative smoking prevalence, as it is unlikely that there is a systematic difference between NRT prescribing rates for veterans and non-veterans other than as a reflection of smoking rates. Veterans with Reserve service only could not be identified from NHS records and have therefore have been included with the non-veterans. This would have had the effect of weakening any observed differences arising as a result of military service. No information was available on the service to which a veteran had belonged (Army, Royal Navy or Royal Air Force); smoking rates are known to differ between the services with Royal Air Force personnel smoking less than those of the other two services [6].

## Conclusion

Veterans born between 1950 and 1954 are at significantly higher risk of lung cancer and other smoking related cancers than their civilian counterparts. This is likely to be due to higher rates of smoking during service and their persistence into civilian life and may be linked to heavier smoking during operational deployment in the mid-1960s and early 1970s. The highest risk is in those with the shortest service. There is no increased risk of lung cancer or other smoking-related cancer among younger veterans, who may have benefited from military health promotion activity aimed at reducing smoking, as well as the overall reduction in smoking prevalence in the general population.

### Ethics approval and consent to participate

Ethical approval was given by the Privacy Advisory Committee (PAC) of NHS Scotland, PAC Reference 27/12.

Individual consent was not required as this was a secondary data study using a pseudo-anonymised database extract.

### Consent for publication

Not applicable.

### Availability of data and materials

The Scottish Veterans Health Study remains in progress and the data are not currently available for access.

## Abbreviations

CI: confidence intervals
ESL: Early Service Leaver
HR: hazard ratio
ICD: International Classification of Disease
NHS: National Health Service
NRT: nicotine replacement therapy
OR: odds ratio
PAC: Privacy Advisory Committee
RR: rate ratio
SES: socio-economic status
SIMD: Scottish index of multiple deprivation
SMR: Scottish morbidity record
